# Health worker migration from South Africa: causes, consequences and policy responses

**DOI:** 10.1186/s12960-015-0093-4

**Published:** 2015-12-03

**Authors:** Ronald Labonté, David Sanders, Thubelihle Mathole, Jonathan Crush, Abel Chikanda, Yoswa Dambisya, Vivien Runnels, Corinne Packer, Adrian MacKenzie, Gail Tomblin Murphy, Ivy Lynn Bourgeault

**Affiliations:** Faculty of Medicine, University of Ottawa, 850 Peter Morand Crescent, Ottawa, K1G 3Z7 Ontario Canada; School of Public Health, University of Western Cape, P. B. X17 Bellville, South Africa; Balsillie School of International Affairs, N2L 6C2 Waterloo, Ontario Canada; University of Cape Town, P/B Rondebosch, South Africa; Department of Geography, University of Kansas, Lawrence, KS USA; East, Central and Southern African Health Community, P.O. Box 1009, Arusha, Tanzania; WHO/PAHO Collaborating Centre on Health Workforce Planning and Research, Dalhousie University, 5869 University Avenue, B3H 4R2 Halifax, Nova Scotia Canada; WHO/PAHO Collaborating Centre on Health Workforce Planning and Research, School of Nursing, Faculty of Health Professions, Dalhousie University, 5869 University Avenue, B3H 4R2 Halifax, Nova Scotia Canada; Telfer School of Management, University of Ottawa, 1 Stewart St., K1N 6N5 Ottawa, Ontario Canada

**Keywords:** Health workers, Migration, South Africa, Human resources for health, Retention, Physicians, Nurses, Pharmacists, Dentists, Policy

## Abstract

**Background:**

This paper arises from a four-country study that sought to better understand the drivers of skilled health worker migration, its consequences, and the strategies countries have employed to mitigate negative impacts. The four countries—Jamaica, India, the Philippines, and South Africa—have historically been “sources” of skilled health workers (SHWs) migrating to other countries. This paper presents the findings from South Africa.

**Methods:**

The study began with a scoping review of the literature on health worker migration from South Africa, followed by empirical data collected from skilled health workers and stakeholders. Surveys were conducted with physicians, nurses, pharmacists, and dentists. Interviews were conducted with key informants representing educators, regulators, national and local governments, private and public sector health facilities, recruitment agencies, and professional associations and councils. Survey data were analyzed using descriptive statistics and regression models. Interview data were analyzed thematically.

**Results:**

There has been an overall decrease in out-migration of skilled health workers from South Africa since the early 2000s largely attributed to a reduced need for foreign-trained skilled health workers in destination countries, limitations on recruitment, and tighter migration rules. Low levels of worker satisfaction persist, although the Occupation Specific Dispensation (OSD) policy (2007), which increased wages for health workers, has been described as critical in retaining South African nurses. Return migration was reportedly a common occurrence. The consequences attributed to SHW migration are mixed, but shortages appear to have declined. Most promising initiatives are those designed to reinforce the South African health system and undertaken within South Africa itself.

**Conclusions:**

In the near past, South Africa’s health worker shortages as a result of emigration were viewed as significant and harmful. Currently, domestic policies to improve health care and the health workforce including innovations such as new skilled health worker cadres and OSD policies appear to have served to decrease SHW shortages to some extent. Decreased global demand for health workers and indications that South African SHWs primarily use migratory routes for professional development suggest that health worker shortages as a result of permanent migration no longer pertains to South Africa.

## Background

Like many countries in Africa, South Africa is reported to face a shortage of skilled health workers (SHWs) [1]. The density of health workers changed little between 2004 and 2013, the most recent years for which data are available. The number of physicians per 1000 population was essentially unchanged at 0.77, the ratio for nurses and midwives experienced a rise from 4.08 to 5.114, and dentists showed a slight increase from 0.13 to 0.2, while pharmaceutical personnel rose from 0.28 to 0.41 [2]. These ratios mask extreme maldistribution between the private and public sectors and urban and rural areas. For example, at the end of 2008, 33 534 medical practitioners (as doctors are referred to in South Africa) were registered with the Health Professions Council of South Africa [3]. But figures, compiled by Health Systems Trust, reflected 11 309 medical practitioners in the public service at the end of March 2010. This translates into only 30% of physicians working in the public sector, the remaining 70% serving the 16% of the population with private medical insurance, and another uninsured 16% who pay out-of-pocket [3]. The country was not considered to have a critical shortage of SHWs according to the World Health Organization Report, “Working Together for Health” [4], and is one of the countries considered to be most likely to achieve a desirable density of skilled health professions [5], although this will depend on the country’s efforts to scale up SHW training. South Africa nonetheless struggles to meet the high demand for health workers in HIV scale-up programs and health facilities and to staff rural facilities [6-10]. Direct financial support for additional SHWs in the public sector from Global Health Initiatives (GHIs) is limited, with most GHIs addressing shortages through training existing health care workers or volunteers to support treatment programs [11,12].

South Africa’s human resources for health (HRH) planning, development, and implementation are best understood in the context of the country’s historical background [13]. Apartheid, the ideology that underpinned racial capitalism, not only compounded inherent inequality in the provision of health care along race, gender, and class lines it also entrenched the development of SHWs along these same lines. The unequal distribution of SHWs favoring the private sector was inherited from the apartheid era, along with underfunding of the public sector. Both legacies still influence today’s supply and mix of health workers. These endogenous problems incentivize and are compounded by actual emigration of SHWs and remain a key challenge for development of the country’s health system. An Organisation for Economic Co-operation and Development (OECD) report on the migration of health professionals showed that, in 2001, South Africa had contributed about 23 400 South African-born “workers practising a medical profession” to just five of the Anglo-American OECD member countries (Australia, Canada, New Zealand, the UK, and the USA), which remain principal destination countries for migrating health workers [14,15]. While this older OECD report remains the “only study presenting detailed data on the migration of skilled health workers from South Africa,” the figures are worrying for their size and consequences of loss of health workers including their future contributions to South Africa as experienced workers over time [16].

Considerable research on the causes and consequences of SHW migration exists, including studies that focus on South Africa [17-23]. Few studies, however, have examined these flows, their consequences, and policy responses to these flows in detail. Comparative studies that do exist have typically focused on macro health indicators, not allowing for a broader investigation of the possible range of implications for patient/population health, providers, and health systems. Further, there has been an almost exclusive focus on medical and nursing practitioners without consideration of other SHWs (such as dentists and pharmacists) who play important roles in health systems. Research to date also has given less attention to the range of policy responses that decision-makers at different levels have either taken or can select to stem the emigration of health professionals or address domestic issues of HRH distribution and shortages [17].

This paper discusses findings of a multi-year comparative study of “source” country perspectives on SHW migration addressing the flows, their causes and consequences, and the policy responses to them.^a^ Four countries participated in this study: South Africa, India, Jamaica, and the Philippines, all known for their high rates of SHW migration [15]. The study posed three overarching research questions:

1. What is the present picture of and recent historical trends in the migration of highly skilled health personnel from South Africa, India, Jamaica, and the Philippines?
2. What, according to those “on the ground” in these countries, are the most critical consequences of the emigration of highly skilled health workers that should be examined, and how could these consequences be measured to optimize the potential for comparative policy analyses?
3. What is the range of program and policy responses that have been considered, proposed, and implemented to address these critical causes and consequences of SHW migration from source to destination countries, and what have been some of the outcomes of these responses?

In this paper, we report on the methods and findings on the causes and consequences of SHW migration from South Africa and the program and policy responses.

## Methods

The study employed a mixed-method comparative approach, comprising scoping reviews of the literature on HRH issues and migration patterns in each country, surveys of SHWs, and key informant interviews. Researchers in Canada (University of Ottawa, Dalhousie University, and Queen’s University) collaborated with researchers in South Africa (University of Western Cape, University of Cape Town, and University of Limpopo) in undertaking the South African component of the study.

### Scoping review

The scoping review of the literature followed the process developed by Arksey and O’Malley [24], using the medical subject headings (MeSH) terms “migration,” “health professionals,” and “South Africa” in a search of PubMed (including MEDLINE) and Embase databases. We also searched the gray literature for documents on migration of health workers using key organizational websites such as the Global Health Workforce Alliance, OECD, the World Bank, the World Health Organization, and the Capacity Project’s Human Resources for Health Global Resource Center. Inclusion and exclusion criteria were used to eliminate studies that did not meet the parameters set by the researchers. Articles were included if they addressed the research questions, mentioned South Africa, and were published between 2000 and 2012.

A database of 301 items was initially collated for South Africa. Applying inclusion and exclusion criteria reduced this number to 189. A full-text review resulted in the exclusion of a further 49 articles, for a total of 140 items retained for the analysis. The research team developed a literature extraction tool to systematically record pertinent aspects of the literature. The literature was analyzed and summarized descriptively and a preliminary report shared and revised with the South African research team.

### Survey

Building on a common survey template designed by the international team, a survey was developed for the health professional groups that were the focus of the South African component of the study: physicians (both generalist and specialist), nurses/midwives,^b^ pharmacists, and dentists. Survey items were constructed building upon previous studies and survey designs (e.g., [25-27]). The South African survey was conducted online over a 3-month period in 2013 and was hosted and carried out by the South African Migration Project (SAMP) and MEDpages, which is a comprehensive private resource of health care contact information in Africa. At the time of the survey, MEDpages had a database of almost 19 000 health professionals for South Africa, including all four health professional groups of our research interest (physicians—including specialists, family physicians, and general practitioners—nurse/midwives, dentists, and pharmacists). Three email broadcasts were made to MEDpages members inviting them to complete the survey. A total of 1623 completed responses were received (see Table 1), a low response rate of 7% but higher than the 2.5% response rate for the 2007 survey [27]. Slightly more than half of the respondents were male, and most were over 35 years in age. Survey data were cleaned and entered into SAS 9 for descriptive and regression analyses.

**Table 1:** **Overview of survey participants**

	**Doctors: generalists (** ***n*** ** = 416)**	**Doctors: specialists (** ***n*** ** = 648)**	**Nurses (** ***n*** ** = 240)**	**Dentists (** ***n*** ** = 141)**	**Pharmacists (** ***n*** ** = 178)**
Sex					
Male	47.36 (197)	57.25 (371)	2.5 (6)	46.1 (65)	35.96 (64)
Female	29.81 (124)	22.84 (148)	69.17 (166)	21.99 (31)	40.45 (72)
Percent non-response	22.84 (95)	19.9 (129)	28.33 (68)	31.91 (45)	23.6 (42)
Age					
25–34	12.02 (50)	5.56 (36)	6.67 (16)	21.99 (31)	13.48 (24)
35–44	22.12 (92)	23.92 (155)	15.42 (37)	14.89 (21)	15.73 (28)
45–54	18.75 (78)	25.31 (164)	28.75 (69)	19.15 (27)	27.53 (49)
55+	22.84 (95)	25.15 (163)	21.25 (51)	12.06 (17)	17.98 (32)
Percent non-response	24.28 (101)	20.06 (130)	27.92 (67)	31.91 (45)	25.28 (45)
Married					
Yes	63.46 (264)	67.28 (436)	52.08 (125)	53.19 (75)	61.8 (110)
No	13.46 (56)	12.96 (84)	20.42 (49)	14.18 (20)	14.61 (26)
Percent non-response	23.08 (96)	19.75 (128)	27.5 (66)	32.62 (46)	23.6 (42)
Years of practice					
1–4	2.4 (10)	2.78 (19)	2.92 (7)	14.89 (21)	2.25 (4)
5–9	12.02 (50)	10.96 (71)	2.92 (7)	19.86 (28)	16.29 (29)
10–19	31.97 (133)	33.8 (219)	20.42 (49)	24.11 (34)	19.66 (35)
20–29	23.32 (97)	25.62 (166)	26.25 (63)	24.11 (34)	34.27 (61)
30+	27.88 (116)	25.31 (164)	41.67 (100)	16.31 (23)	25.28 (45)
Percent non-response	2.4 (10)	1.54 (9)	5.83 (14)	0.71 (1)	2.25 (4)

### Key informant interviews

Interviews were conducted with a range of key informants including but not limited to professional educators, regulators, national government agency officials (e.g., dealing with immigration and HRH), local government authorities, private and public sector health facilities, recruitment agencies, and representatives from professional associations and councils (Table 2). Purposive sampling was used to recruit key informants using three criteria: (i) their organization’s active role in SHW migration-related issues, (ii) their position within the organization (sufficiently senior to speak to the issues), and (iii) their experience related to the research questions we were exploring. Formal invitations were sent via email and included a consent form, contact details for project coordinators in South Africa, and the interview questions. Some questions were asked of all informants, while specific questions were developed for different types of organizations to allow for deeper probing. Respondents were also asked to suggest additional candidates for interviews (snowball sampling).

**Table 2:** **Distribution of key informants by stakeholder group**

**Stakeholder group**	**Number of participants**
Education/training institutions	12
Private sector	7
Public sector	3
Professional regulatory boards	5
Professional/worker association	7
Development partners	2
Recruitment agencies	2
Provincial government	3
National government	2
Returned migrants	2
Total	45

Interviews were conducted between May 2012 and June 2013 by four members of the research team, two from the University of Western Cape and two from the University of Limpopo. Interviews were 45–90 min in length and were conducted either in person or by telephone. All interviews were digitally recorded, transcribed, and reviewed by data analysts to ensure accuracy of information gathered. In addition, detailed notes were taken during the interview, and part of that information was used in subsequent interviews to ensure data gathered were informed by and built upon previously collected data.

### Data analysis

Quantitative data collected from the surveys was analyzed descriptively as well as with regression models using SAS 9 software. Three logistic regression models were used—one each with respondents’ self-reported application (or not) to (1) write the licensing exam for their profession to practice in a foreign country, (2) obtain a work permit to practice in a foreign country, and (3) obtain a permit to live in a foreign country as dependent variables. Independent variables included respondents’ profession, sex, age group, and marital status. Qualitative data from the key informant interviews were analyzed thematically using ATLAS.ti software following an initial comparative coding structure developed by the international team and augmented with emergent codes derived from the interviews themselves.

### Ethics

Approval to conduct the study was received from the University of Ottawa Research Ethics Board and those of the University of Western Cape, University of Cape Town, and University of Limpopo. Further permissions for the KII component of the study were obtained from the health departments of the South African provinces and the health institutions in which interviews took place.^c^

## Results

### Who is migrating?

All four SHW categories (physicians, nurses, dentists, and pharmacists) included in the online survey conducted for this study continue to emigrate from South Africa, although there has been an overall decreasing trend in out-migration since the early 2000s [28]. In the late 1990s, there was a significant increase in outward migration of nurses from South Africa, mostly to the UK [23,29]. In 2001, the total number of nurse emigrants was roughly equivalent to 20% of the total number working within the public sector in South Africa. In 2000, South Africa was the 8th of 10 top-ranked countries for numbers of emigrating physicians, at 4400 [22]. More recent estimates of South Africa-born physicians working outside the country in comparison to counts of those who work in the country range between 21% [19] and 29% [18], although not all émigré physicians completed their training in South Africa [22]. As for many countries, the Government of South Africa does not collect specific records of skilled health worker migration. Estimates are often based on information from licensing bodies and immigration records. Additionally, there is no regional repository of data on labor migration [30].

Interviewees broadly agreed that out-migration of SHWs has slowed in recent years, particularly to some of the former preferred “destination” countries. Physician emigration to the UK, for example, was described as having “slowed considerably due to …… the introduction of the examinations to enter the UK,” (K004, Academic director) a condition for licensing not previously required. According to the experience of one senior physician and professional association representative, there have been no recent examples of emigration:

The last I recall was in 2011 when we lost a doctor from our oncology unit to Canada; at about the same time we lost another doctor from Ukraine, but that one went back to his home country. (K032, Professional association representative)

A former high official from the National Department of Health observed:

Of course it has gone down dramatically, now as a country we breathe a sigh of relief. Not so many people are going out now. (K031, Senior Government Department of Health official)

Although the “pull” factors may have moderated somewhat as a result of fiscal austerity in “destination” countries, medical “brain drain” from South Africa is unlikely to subside significantly in the short- or medium-term as uncertainty mounts concerning new policies, such as the introduction of a National Health Insurance scheme and its possible impact on the private health sector. As one interviewee noted:

If you look at three years ago, there was a huge exodus of doctors. I think it is being settled now… but the outflow is still there and I think the biggest negative influence at this stage is the NHI [National Health Insurance]…[Physicians] haven’t got information [on] the impact that it will have, so they run away before the fire starts. (K013, Private hospital group executive)

Nurse migration from South Africa is similarly regarded as declining, partly attributed to a slowdown in recruitment from the UK since 2006 when new immigration rules allowed foreign nurse work permits only if employers could demonstrate non-availability of staffing from Britain and European Union countries [31]: “you don’t hear much of [nurses] going to London” (K011, Nursing school representative). While active recruitment from the favored Anglo-American destination countries has fallen in recent years [20,32], it continues from a new group of transit countries: “Saudi Arabia, the UAE, Oman [and] other countries in the Gulf region, they still recruit nurses” (K008, Academic director).

The primary reason for the perceived slowdown in nurse migration, however, is likely the result of domestic policy changes, notably the Occupation Specific Dispensation (OSD), discussed later in this paper [16]. Although one informant discussed how the OSD had “stopped” (sic) the flow of internal nurse migration from the public to the private sector, new concerns have arisen. These include questions about overall nursing adequacy related to domestic private hospitals “poaching” from each other or nursing staff being lured from the public sector to work in better resourced programs funded primarily by global health initiatives, especially those pertaining to HIV/AIDS. Recent research suggests that “moonlighting” (having a second job in addition to a primary job) is a predictor of nurses’ intention to leave South Africa [33].

There has been little recent detailed study of migration by dentists and pharmacists. Health professionals other than doctors and nurses tend to be included in a category described as “other health professionals” [34]. Interviewees in our study noted that for dentists, migration “seems to happen in waves… [t]here is a time when there is an increased movement then it settles down for a while” (K007, Academic director). For pharmacists, there was an initial surge in out-migration immediately post-democracy (1994), but this dropped as fewer overseas positions became available due to increased supply in destination countries while, within South Africa, “the opportunities are too many now for people to want to go” (K018, Hospital pharmacy manager).

**Figure 1:**
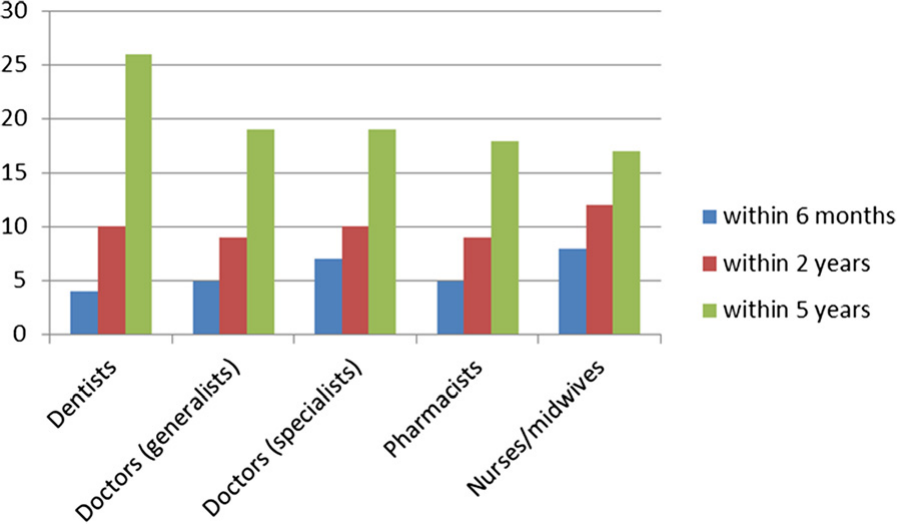
**Percentage of respondents reporting “very likely” to migrate by profession and time frame.**

Some of these perceptions were corroborated by the survey results, with only 10% of the survey sample reporting that they were very likely to migrate within the next 2 years. However, this migratory intent varied by professional group, and the figure was much higher when a 5-year future was considered (Figure 1). If the “somewhat likely” response is added to the “very likely,” half of all doctors and pharmacists, 60% of dentists, and just over 40% of nurses reported some likelihood of seeking work in another country within the next 5 years. Nurses showed the highest likelihood to migrate within the next 2 years. Almost half of health professionals said they were more likely to consider migration now, than 5 years ago, and “often” or “sometimes” sought migration information through professional sources.

**Figure 2:**
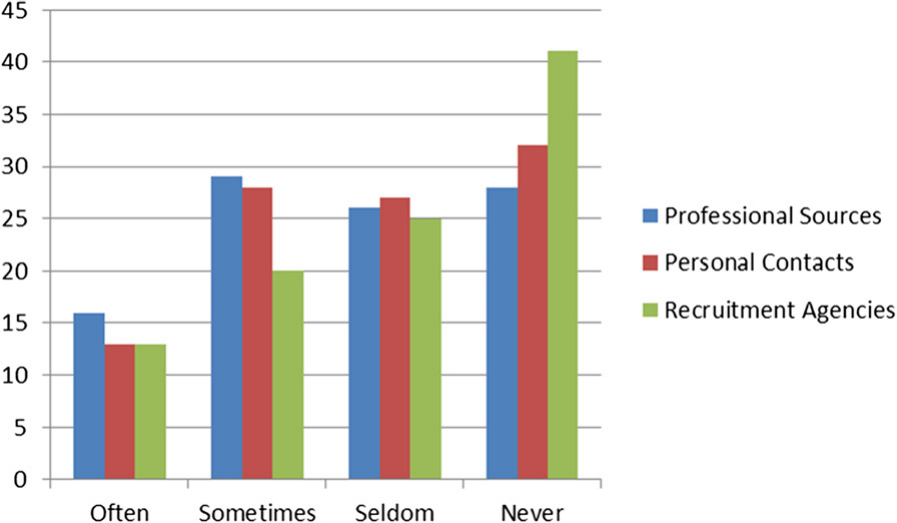
**Percentage of respondents seeking migration information by frequency and source.**

**Figure 3:**
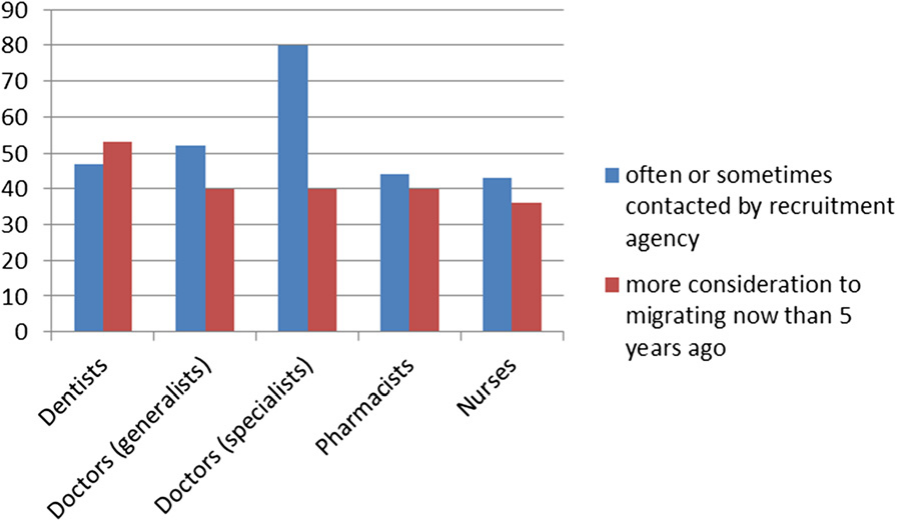
**Contacted by recruitment agency and migration consideration.**

Few reportedly sought no information at all, indicating that migration continues to be a consideration (Figure 2). Although recruitment agencies are not seen as a major source for migration information, almost half or more of our sample across the different professions (in the case of specialist doctors, 80%) were contacted often or sometimes by recruitment agencies operating in South Africa, indicative of some active recruitment still occurring (Figure 3).

Regarding actual intention to migrate, when participants were asked whether they had applied for a foreign work permit, foreign residence, or a foreign license, our regression model (which controlled for age, sex, and marital status) revealed that pharmacists were significantly less likely than nurses to apply for a work permit, generalists were more likely than nurses to apply for residence (though this was significant at the *P* < .05 level), and both generalist and specialist physicians were two to six times more likely to apply for a license than nurses. Interestingly, female health professionals were 1.5 times less likely to apply for a work permit or for a foreign license than male health professionals across all groups.^d^

### Return migration

The survey included questions for respondents who may have been practicing in another country but who have since returned. One third of survey respondents indicated that they were return migrants, suggesting that health worker migration from South Africa was beginning to resemble the “circular migration” commonly discussed in the literature [35,36]. There was considerable variation among the professional groups, with specialist and generalist physicians more likely to be returnees. A fifth of the nurse respondents indicated that they had practiced abroad, but too few dentists answered this question to report. Most physicians and nurses were returning from work in the UK, much of it short-term (<3 years).

Major reasons cited for return migration were South Africa’s physical environment, family ties, and South African “lifestyle,” “culture,” and “social life,” while cost of living and cold weather were the big “pushes” out of their destination countries.

Key informants similarly noted a return migration phenomenon. As one head of medicine noted with respect to doctors:

They are using the option of going [abroad] for a few years to earn some money… not with the idea of settling down permanently but gaining experience and earning some money and then coming back, setting up a practice here [with] a nest in which to work. (K020, Hospital medical head)

Despite the low numbers of returned pharmacists in our survey, interviewees noted that “the trend is [pharmacists] wanting to come back to South Africa… from the UK in particular” (K018, Hospital pharmacy manager). Most returning pharmacists are entering the public sector. A similar trend was reported locally with pharmacists moving from the private to the public sector, attributed to the introduction of OSD.

**Figure 4:**
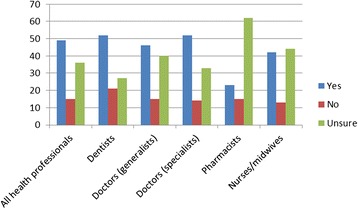
**Percentage who consider their return to be permanent by profession and response.**

While the extent of return migration does appear to be a novel finding of our study, the evidence of the permanence of such a return is more equivocal (Figure 4). Fewer than half of returned health professionals considered it permanent, and although only a small portion is definite that they will likely leave again, many remained uncertain.

### Why are health professionals migrating?

The uncertainty of a permanent return leads to a re-consideration of why South African health professionals consider migration in the first place. The reasons for skilled health worker (SHW) migration in general have been well documented and discussed in the literature, often using the simplistic but descriptively intuitive “push”/“pull” dichotomy. Push factors drive outward migration and, for South African SHWs, include low remuneration, poor living and working conditions, lack of career development opportunities, high burden of HIV and MDR-TB, high cost of living, and job and economic insecurity [20,37-45]. Pull factors draw inward migration to higher income destination countries and generally include the availability of positions, higher remuneration, better living and working conditions, career development opportunities, and promise of safety and security for the family [20,39,43,46-50]. Active recruitment by destination countries can work in combination with both “push” and “pull” factors [32,51].

In a comparison of our 2013 survey with a 2007 SAMP survey [27] which was conducted using similar questions and methods with physicians, dentists, and pharmacists, health professionals’ satisfaction with several aspects of their working conditions had improved, particularly in the ability to find a desirable job, workplace infrastructure and morale, job security, prospects for professional advancement, workload, and income levels [52]. Nurses in our 2013 survey (who were mostly from the private sector) reported some satisfaction with their workplaces with one exception: they were less likely to report satisfaction with their income level, a complaint also reported more frequently by dentists than by doctors or pharmacists.

Generally, one would expect positive changes in working conditions to reduce the “push” to migrate. Although slightly fewer doctors, dentists, and pharmacists reported some likelihood of migrating in 2013 than in the 2007 survey, over a longer term, almost half think they will leave South Africa, reflecting that, while workplace satisfaction may be improving, considerable dissatisfaction persists. An expressed intention to migrate does not necessarily mean that a SHW will eventually leave, but the scale of this intention contrasts starkly with the comments by our key informants on the marked decline in SHW migration. Push factors are also shifting somewhat from workplace concerns to broader political and economic ones. Dissatisfaction with affordable goods and cost of living is high; fewer than 10% reported being satisfied with their personal or family’s safety or their children’s future, and almost none of the respondents were satisfied with the country’s high levels of poverty and inequality.

**Figure 5:**
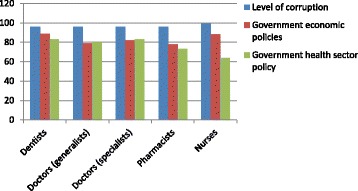
**Percentage of respondents dissatisfied by profession and government action.**

Even recognizing that it is easier to find fault with government policies than praise, the low levels of satisfaction with key measures of government action are striking (Figure 5). Nurses and pharmacists are less dissatisfied (more satisfied?) than dentists and doctors, particularly with government health policies, notably the OSD. But the universal concerns over corruption and economic policy are consistent across professions and indicative of potential reasons for migration. This is confirmed by another survey question, asking respondents to identify the three most important reasons for wanting to migrate in each of two categories—living and working conditions (Table 3). What is apparent is the high level of dissatisfaction with the state of politics and governance in the country with a fear for personal safety and what the future holds for their families, especially children. That more living conditions were rated “very important” by respondents than were working conditions suggests a shift in what had traditionally been “push” factors, a result confirmed by our key informant interviews.

**Table 3:** **Top-rated reasons for wanting to migrate**

**Category**	**Reason**
Living conditions	1. Level of corruption (83%)
	2. Personal and family safety (81%)
	3. Children’s future in South Africa (80%)
Working conditions	1. Lack of respect from government (60%)
	2. Poor infrastructure, supplies (55%)
	3. Personal security in the workplace (54%)

Our key informants expanded on these themes, noting that for many, and notably return migrants, “a lot of it is finances” (K004, Academic director), a desire to quickly amass savings to bring back home (for nurses) or to pay off quickly student loan debts (for doctors).

There was also a strong indication of push factors at the work place for nurse migration:

Most of them complained of conditions of work - accommodation, equipment, resources, tools of trade and management. And money of course comes in, but not as first issue; we all want to be satisfied with what we do, otherwise why wake up in the morning to go do what you don’t enjoy. So poor job satisfaction because of all that nonsense at work was the issue. (K024, Academic director)

Domestic policies play a role, especially for dentists since dental care costs are paid out of pocket and as a result many people seeing a dentist only every 2 or 3 years. Similar to the survey findings, the biggest push identified by informants was “political instability in the country… that was a major drive” (K020, Hospital medical head).

### What are the impacts of health worker migration?

One of the positive contributions claimed for SHW migration from South Africa is the new skills brought back when they return.

One of the good things about those that go is they…do continuing courses…so when they come back they are better skilled than when they went. (K007, Academic director)

That’s another aspect of exposure, some ways of doing things maybe better ways of doing, maybe exposure to some other types of technology. (K008, Academic director)

Remittances are the other primary benefit, claimed by some economists to offset any of the negative losses associate with SHW migration [53,54]. Of all eastern and southern African countries, South Africa is thought to receive the second highest amount of remittances (US$ 1 billion) after Kenya (US$ 1.8 billion) [22]. Remittances, however, do not directly benefit the public health or the educational system (apart from whatever government taxes might be levied on them) and are consumed often for basic necessities, benefitting only individuals, families, and households [38,55]. As one key informant commented, “Yah you know, somebody would say for instance ‘I’ve started improving my home’ so she is remaining there (out of South Africa) so she can finish up the project” (K003, HR executive). The extent and the intent of migrating South African SHWs to remit is largely speculative. A recent survey of South African physicians in Canada found low rates of remittances, with only 19% remitting regularly (monthly) and nearly 30% never remitting [56].

**Figure 6:**
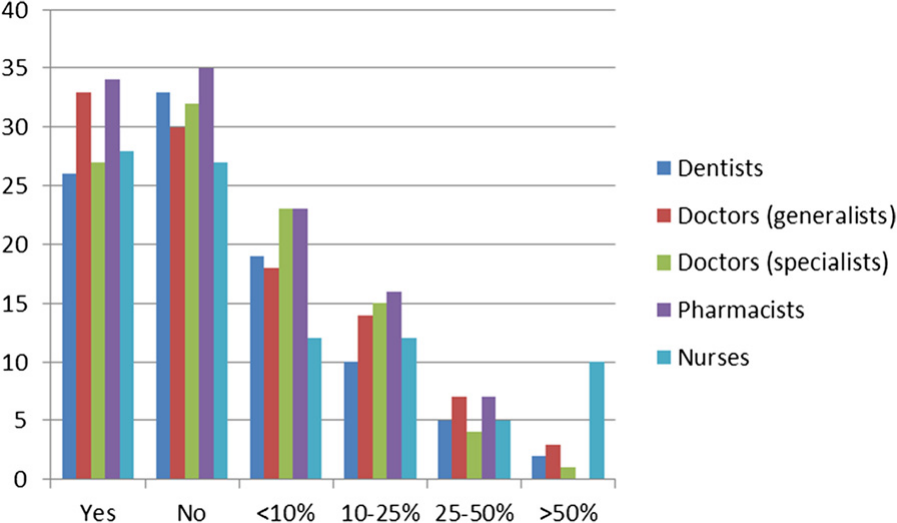
**Percentage of respondents expressing likelihood to remit and by percent of foreign earnings.**

Our survey asked about the intention to remit after emigrating and the anticipated portion of their foreign earnings they would send home (Figure 6).

Although many respondents were unsure if they would migrate, or uncertain whether or not they would remit, for those with a more definite intent to migrate, the findings indicate that slightly more were planning not to remit. Of those planning to remit, the frequency fell as the remittance amount rose. Compared to other professional groups, nurses plan to remit smaller portions of their overseas earnings (perhaps owing to lower wages against cost of living in destination countries) but are more likely to remit over half of their incomes. There appears to be little support for the argument that remittances offset the loss of skills, experience, or investment in training. Based on an analysis of lost return on investment, the cost to South Africa of émigré physicians alone has been estimated at $1.41 billion [57].

Despite some positive effects, the impact of SHW migration is perceived as largely negative. A prime concern is staff shortages. Such shortages partly arise from poor management of an under-funded public health system, where unfilled posts for physicians rose from 30% in 2006 to 49% in 2010; the parallel figures for nurses were 31% and 46%, respectively [58,59]. Despite a small increase in the number of health professionals being trained in the country and electing to work in the public sector, most medical graduates specialize, enter the private sector, or emigrate [52]. This is occurring even as there are projections for a decline in the doctor-patient ratio between 2010 and 2020 [52]. Internal SHW movement remains a concern, with the private sector said to be “poaching” doctors from the public sector, which in turn is poaching nurses from the private sector. Many SHWs in the public sector also migrate to GHIs; the President’s Emergency Plan for AIDS Relief (PEPFAR) alone in 2010–2011 was paying the salaries of 20 000 SHWs in NGOs providing HIV treatment and prevention programs; also, working conditions are often better in GHIs than in the public sector [52].

Too few staff to meet care demands leads to “burnout” and high turnover of staff [60]. An older survey of nurses found that 89.6% of the respondents were considering leaving South Africa because of the heavy workloads [61]. Job stress was found to be higher among South African physicians than physicians working in Europe or the USA, caused by working overtime; making critical, on-the-spot decisions; and dealing with crisis situations [60]. Despite some improvements in working conditions for South African SHWs, staff shortages and burnout persist: “I’m an intensive care nurse myself, and I’ve had to work a 48-hour shift before, because of [shortages]” (K019, Private hospital group executive) with knock-on effects for patients: “So we often close our casualties, we close for the ambulances to by-pass because we don’t have staff” (K019, Private hospital group executive). A nursing association official indicated:

Those who remained worked even more with more patients. And you had to think - do the people here think I am not good enough to also go abroad? That kind of thing which affected the morale and interest of nurses. I know some could not go so they left nursing altogether. That’s how bad it was then. (K030, Nursing association representative)

Outward migration exacerbates these domestic shortcomings. It is particularly acute for some professions, as a head of a pharmacy school lamented: “It’s a loss for us. If we had all the pharmacists who had left the country, we probably would not have the shortage. There was a time when we lost 1000 a year” (K006, Academic director).

Even with evidence of return migration, the number of SHWs falls well short of what is needed and linked, at least in part, to the impacts of migration. The private sector is not exempt from shortages. The specialist to patient ratio was so bad in one private hospital group that “it is one specialist, one paediatrician for every 30,000” (K013, Private hospital group executive). This executive complained that “it’s unacceptable, but still they poach our paediatricians and take them abroad” (K013, Private hospital group executive), another indication that active recruitment has not entirely disappeared. Defending its physician recruitment from the public sector, one private sector marketing manager argued that it was better to have doctors work in the private sector than to leave the country, the implication being that it is public sector conditions that push physicians out.

The impacts of SHW migration affect training institutions as well:

The consequences are really gross [sic] because, for example, right now in our department there are only three of us who are doctoral-trained [and] we have quite a number of postgraduate students that we have to handle. (K011, Nursing school representative)

Despite continuing pushes and pulls for SHW migration, key informants were more concerned with the domestic management of health professionals than with out-migration per se. Migration was no longer seen as the primary problem facing the country’s health system. At the same time, in response to a survey question on the effect of SHW migration on the country’s health system, over 90% of doctors and pharmacists, and over 80% of dentists and nurses in the survey sample, considered out-migration to have a somewhat or very negative impact, yet there is little evidence that this view is dissuading individual professionals from leaving the country. Only slightly fewer were concerned with the negative effects of rural to urban SHW flows, while roughly two thirds believed that public to private sector migration within the country also disadvantaged the health system.

### What policies have been introduced to mitigate these impacts?

Several multinational, bilateral, and domestic strategies have been pursued to mitigate the negative impacts of SHW migration (Table 4). The Commonwealth Code [62], forerunner to the WHO Code, was an agreement among Commonwealth countries (including South Africa) on ethical recruitment. It proved ineffective in managing migration due to its voluntary nature and its focus on public recruitment to the neglect of private recruitment [63,64]. The Health Worker Migration Initiative (OECD) led to formulation of the WHO Global Code of Practice on the International Recruitment of Health Personnel [65], which, like the Commonwealth Code, has been hampered by its voluntary nature with a very low degree of reporting on Code implications by WHO member states (Bourgeault, Labonté, Packer, Runnels, and Tomblin-Murphy (forthcoming)).

**Table 4:** **Some strategies to mitigate health worker migration and address health worker shortages in South Africa**

**Scope**	**Strategy**
Global agreements	• Commonwealth Code (2003)
	• Health Worker Migration Initiative (2007)
	• WHO Global Code of Practice on International Recruitment of Health Personnel (2010)
Domestic policy statements on migration	• South Africa’s Policy statements on health worker immigration (2001; 2006)^a^
Bilateral agreements	• UK/South Africa MOU (2003)
	• Cuba (1996)
	• Germany
	• Tunisia (1999 and 2007 technical agreement)
	• Iran 2004
	• US PEPFAR/Medical and Nursing Education Partnership Initiative (2013)
Destination country agreements with potential to impact South African migrants	• UK NHS Code of Practice on the Ethical Recruitment of Health Professionals (2004)
South African initiatives to prevent migration by improving health system human resources for health and living and working conditions for health workers	Examples include
	• Increasing SHW production
	• Student Sponsorship Programs
	• Community Service Program
	• New SHW cadres
	• Task-shifting
	• African Health Placements
	• Occupation Specific Dispensation (OSD)

^a^South Africa has added policies limiting recruitment. In 2001, South Africa stated that it would not recruit HHR from Commonwealth and G77 countries [34]. Furthermore, South Africa agreed it will not recruit health care professionals from developing countries or Organization of African Unity countries [69].

#### Domestic policy statements on migration

South Africa is not just a source country for SHWs, but it is also a destination country. Almost a quarter of physicians in South Africa are foreign-trained [41], the majority of whom (78%) work in rural and remote areas. Regionally, South Africa is party to the Southern African Development Community (SADC) Treaty 1992 and its Protocol on the Facilitation of Movement of Persons, signed in 2005 [66] to regulate the legal movement of persons in the region [67-69]. This Protocol, however, is not yet in force as too few SADC Member States have ratified it to date, although South Africa has undertaken its own stance on the issue [70]. In 2001, it stated that it would not recruit foreign health workers in the SADC region except under intergovernmental or bilateral agreements. In a 2006 Policy statement, it stated that “recruitment of individual applicants from identified developing countries, in particular from another SADC country, will not be endorsed by the Department”. As an official from the Nursing Council noted, “In terms of this [SHWs] arrangement, we can’t fish from the same pond” (K017, Regulatory body representative). According to some key informants, this policy has not prevented individuals from SADC migrating to and being given work permits in South Africa, suggesting ready movement across Southern African borders into South Africa.

#### Bilateral agreements

South Africa recruits from non-SADC African countries, as well as from Europe, North America, and India. It also has bilateral agreements with Tunisia, Iran, and Cuba that allow it to bring in doctors from these countries. The oldest and most successful bilateral agreement has been with Cuba, which has a deliberate excess supply of physicians. Close to 500 Cuban doctors were working in South Africa at any one time, their remittances an important source of foreign currency and taxable public revenue for Cuba [38]. Although never a substantial portion of the SHW labor force, most Cuban doctors worked in rural and underserved areas [41,71]. While the deployment of Cuban doctor “brigades” in South Africa appears to have all but disappeared, training of South African medical students in Cuba has expanded from approximately 1200 in 2013 to over 3000 in 2015 [72,73]. South Africa has sent primarily black and disadvantaged high school graduates, many of them from rural areas, to attend medical training in Cuba. Students receive full scholarships with an expectation of return to practice in the public sector in South Africa for the amount of time spent in training in Cuba (5 to 6 years) [74].

The US-funded PEPFAR program since 2013 has also supported Medical Education and Nursing Education Partnership Initiatives [75,76]. To date, two South African medical schools (University of KwaZulu-Natal and Stellenbosch University [77]) have benefited with funds to recruit and train students from rural areas to increase chances of retention after they graduate, and plans to improve nursing colleges also exist [78].

As well as between-country agreements, there are between-university agreements:

There are also agreements in place between university departments in South Africa and those in other African countries and they sign a Memorandum of Agreement not to recruit doctors they train in South African universities. (K020, Hospital medical head)

Despite the contributions that immigrant skilled health workers make to South Africa, our survey respondents generally reported low rates of satisfaction with their presence in the country.

#### South African initiatives

The most promising initiatives to mitigate health worker migration and associated shortages of health workers are those undertaken within South Africa itself through the improvement of health system human resources for health and living and working conditions for health workers. There have been efforts to increase training of SHWs notably with increased intakes for nurses, an increase in the number of private nursing colleges, and a commitment to re-open 84 colleges which are inactive while renovations are underway. A new medical school will open in Limpopo in 2015, the ninth in the country [79], although training posts in the country’s medical schools are often vacant due to funding shortfalls [52]. A number of training institutions now work with provincial governments in efforts to retain staff in rural locations. Strategies include sponsorship of some students who will then be required to work in the respective sponsoring provinces for several years after they finish. As well, an academic director of one university said that they “invite provincial coordinators to speak to the students…to encourage them to apply for community service in their provinces” (K007, Academic director), explaining the leisure, allowance, and other benefits of such service provided by the provinces.

**Figure 7:**
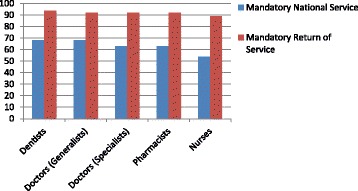
**Percentage of respondents by profession who think mandatory national or return of service justified.**

Relying upon community service requirements to stem migration has had an episodic history in South Africa [80]. Surveys of such “bonded” physicians have found that almost a quarter would leave the country due to the poor working conditions [81]. The 1-year requirement of such community service as a condition for registration provides only temporary relief from physician shortages at best. Our survey asked respondents whether mandatory national service or specific return of service (when students received a government bursary for their training) was justified, and a number consistent across all professions agreed that it was. One exception was a lower rate of agreement by nurses for mandatory service (Figure 7). At the same time, almost a third of all respondents agreed that mandatory national service would actually make them more likely to migrate, compared to a very small number who saw such a requirement as a disincentive to leave. This challenges any consistent policy interpretation.

Another domestic response has been to introduce new cadres of skilled health workers such as clinical associates (Clin-As) and pharmacy support personnel (technical assistants and pharmacy technicians). These workers are sufficiently skilled to meet South Africa’s particular needs but whose qualifications, because they are specific to South Africa, would be less likely to be recognized by destination countries [82-85]. Training of less skilled workers, such as community health workers, is also being piloted in several provinces. Task-shifting, such as nurse-initiated and managed antiretroviral therapy, is also expected to reduce some of the migration-incentivizing burdens on generalist and specialist physicians [83].

The African Health Placement (AHP) Program, which seeks to attract SHWs to South Africa for short- or long-term stays, has liaison officers to market the program in the UK, Canada, and the USA. The aim is to increase supply in rural areas, but given the difficulty of recruiting for such areas, placements are first offered in semi-urban areas [86]. Although “it has been a strategy of AHP to try and target South African doctors who feel lost and lonely overseas, it hasn’t been really successful, it’s been small numbers” (K004, Academic director). One reason for the lack of success is competition from other recruiting countries with much larger budgets, such as Australia. More success is reported attracting other overseas African physicians to South Africa:

One of the advantages of your African doctors coming in here is that they have to write exams so they end up staying here for longer…The majority of them that come into the country actually eventually immigrate here. (K001, Public sector recruitment specialist)

The biggest recent change in South African policy, however, has been the OSD Program. Introduced in 2007, the OSD is a financial incentive strategy to attract and retain SHWs in the public health sector. Nurses were the first to benefit from the OSD in 2007, while salary adjustments for other health professionals did not take place until 2008–2009. Despite critiques of the program [87], the OSD has narrowed the gap between salaries at home and abroad [88] and the OSD has been cited as one factor that appears to have slowed out-migration and promoted return [89], especially for nurses who registered significant improvements in their pay and benefits.

With the introduction of OSD, we found some people coming back especially with the nurses because they were there in numbers. We found them coming back because… you know when OSD was introduced, it considered the previous experience, even the experience you had overseas so at least it was easy [for nurses] to come back. (K003, HR executive)

Although salaries for generalist and specialist physicians also increased substantially when their OSD benefits came on line, support from doctors has not been as positive as from nurses, attributed to poor consultation in the development of the program [52].

As some of our informants noted, what may prove even more significant than the OSD program in terms of SHW decisions about whether to stay or to leave is the slow rollout (estimated at 14 years) of a National Health Insurance (NHI) program for the country, first announced in a 2011 Green Paper [90-92]. Given these delays, some doctors’ apparent resistance [93,94] to work at clinics in pilot districts, and failures to improve the public health system and infrastructure—all required for the implementation of the NHI scheme—make it difficult to assess the following: the novelty of this program precludes any analysis or commentary of its possible implications on SHW migration choices. Further, there is little in the way of evaluation or evidence of impact of any of the above strategies designed to mitigate health worker migration from South Africa.

### A note on study limitations

Some limitations have affected the study. Most immediate of these is the lack of availability of recent, systematically collected, and disaggregated formal data on the migration of SHWs to and from South Africa. This precludes researchers from a more rigorous assessment of the extent of such migration, the determinants of migration, and its impacts and consequences. The South African government has instituted a number of policies to improve its health systems and working conditions for HRH, thus discouraging health workers from leaving. In general, these policies are intended to benefit all SHWs including any intending to migrate. The cross-sectional nature of the study is another limitation, as it does not permit analysis of SHW migration over time, nor in relation to domestic policies or other contextual and global influences with potential to impact migration. At the time of writing, our information suggests that migration by SHW s from South Africa has diminished, although this is difficult to demonstrate quantitatively as a result of data limitations.

Another limitation is that although the private sector is a significant player in South Africa, our data collection and interpretation has emphasized public sector migration causes, impacts, and policy responses. There is some evidence of the movement of health workers from the public to the private sector, but we were unable to determine how and if private health care in South Africa is a contributing source to health worker emigration. Further studies of the relationship of the private and public sectors, the role of moonlighting in health worker retention, and internal movement of skilled health workers would assist in this determination. A limitation in our key informant sampling was the small number of private recruitment agencies, many of which were reluctant to be interviewed. Determining these actors’ influence on skilled health worker migration would be helpful for nuancing the concepts of passive and active recruitment.

Our survey response rate of 7% from a database of nearly 19 000 health professionals which included physicians, pharmacists, nurses, and dentists is considered low for a targeted survey. As the survey was delivered through the Internet, those without access to the Internet were excluded from the survey. Those who were particularly disgruntled and fully intending to migrate may have found greater reason to return the survey, hence a possible bias in the survey’s findings. Also, the response for nurses was very small for the size of the MEDpages database, and most of the respondents were private sector nurses (personal communication, J. Crush).

## Conclusion

Despite some evidence that SHW migration from South Africa is not as prevalent as when it reportedly peaked in the late 1990s and early 2000s, the intention of health professionals to migrate remains very common. Data from our survey suggest a similar level of intention to migrate, and less dissatisfaction, compared to that found in a 2007 survey [27]. This is despite improvements in compensation for most health professionals in the interim. Levels of dissatisfaction with South Africa’s economic and political situation reported through the survey are extremely high. Corruption concerns top the list, along with a fear for personal safety and what the future holds for their families, especially their children. Workplace “push” factors also persist, although once more these circle back to government policies, notably a feeling of disrespect by government and poor infrastructure.

The salary and other benefit improvements of the OSD program in the public sector is credited by some key informants with slowing down out-migration, in contrast to the limited impact of most international and global codes on recruitment. Despite nurses being perceived as benefiting most from the program, and expressing the least dissatisfaction with government health system policy, they remain the category most likely to report intentions to migrate in the short-term, least supportive of mandatory national service, and least satisfied with their level of pay. These findings cluster into a pattern in which nurses are more likely to initiate short-term out-migration, earning money for investment back into their families and households.

Although active recruitment is perceived to be less prominent than in earlier years, there is evidence that it persists. Nurses in particular are reported to be actively recruited by the Gulf States. Given the global reach of communication technology, active recruitment likely plays only a small role in a health professional’s decision to migrate. If a person feels compelled to leave, and positions are available, the brakes on out-migration are primarily those associated with family ties, yet it is concern for the security of one’s family and children that ranks high as a reason to migrate in the first place.

One compelling finding from our study is the large number of SHWs who are return migrants to South Africa. Again, the OSD is cited as an incentive for such return, while the AHP intended to entice émigré health professionals to return is thought to have largely failed. Although circular migration—a key issue in the international literature on skilled worker migration—does appear to be common, it does not appear to be permanent. Excluding pharmacists, for whom the OSD and public sector employment now appears to be a draw, almost half of return SHW migrants are likely to leave again in the near future. Many return migrants are simply uncertain whether they will remain in South Africa, with improvements (or deteriorations) in South African working and living conditions likely to be the deciding factors. Most consider skilled health workers’ departure to have a negative effect on the country’s health system, yet that does not appear to have an influence on dissuading them from leaving. Most also believe that mandatory national or return-of-service requirements would be justified, yet almost a third considered such requirements would make them more, rather than less, likely to migrate. This places such policy options between the proverbial rock and a hard place, unless more intelligence on how such requirements could incentivize SHWs to remain in the country was gathered.

A novel finding from our study concerns the intention of émigré SHWs to remit portions of their foreign earnings. Of those who responded to this survey question, slightly more expressed no intent to remit than those who would. The amounts those planning to remit were also quite small, implying that remittances are highly unlikely to compensate for the losses (both direct training and indirect investment returns) generated by their departure. The rhetoric of return or circular migration may have some substance based on our study’s findings, the countervailing argument emphasizing the value of émigré remittances does not.

There are two outstanding issues that require more attention. The first is the role of South Africa as both source and destination for SHWs, its reliance on foreign-trained physicians from other parts of Africa (and elsewhere), and the apparent antipathy of its domestically trained SHWs towards foreign health professionals. The second is the impact of the still-nascent rollout of the country’s national health insurance scheme. There is some concern that this could spark at least a short-term exodus of health professionals, especially generalist and specialist physicians, due to uncertainty of the implications of the program for their future practice, especially in terms of private sector opportunities.

If there is a single take-home message from this study, it is this: It is impossible to disentangle the “pushes” behind migration from how health workers perceive or experience South Africa’s health care system specifically and the political and economic context and policies of the country more broadly. The latter now figures prominently in health workers’ intentions to migrate.

## Endnotes

^a^“Source Country Perspectives on the Migration of Highly Trained Health Personnel: Causes, Consequences and Responses”, Canadian Institutes of Health Research (CIHR) study (MOP 106493).

^b^In South Africa, most registered nurses are also midwives.

^c^University of Ottawa Ethics Approval Certificate numbers H07-10-02H; H07-10-02C (Limpopo and University of Western Cape; H07-10-02E (University of Cape Town) for the Canadian Institutes of Health Research (CIHR) funded study, “Source Country Perspectives on the Migration of Highly Trained Health Personnel: Causes, Consequences and Responses”, (MOP 106493).

^d^Reasons for these gender differences were not explored through the survey but through the key informant interviews.

## Abbreviations

AHP: African Health Placement (Program)
GHI: Global Health Initiative
HRH: Human resources for health
NHI: National Health Insurance
OECD: Organisation for Economic Co-operation and Development
OSD: Occupation Specific Dispensation
SADC: Southern African Development Community
SHW: Skilled health worker
